# Procedure for optimal implementation of automatic tube potential selection in pediatric CT to reduce radiation dose and improve workflow

**DOI:** 10.1002/acm2.13098

**Published:** 2020-12-18

**Authors:** Jacinta E. Browne, Michael R. Bruesewitz, Vrieze Thomas, Kristen B. Thomas, Nathan C. Hull, Cynthia H. McCollough, Lifeng Yu

**Affiliations:** ^1^ Department of Radiology Mayo Clinic Rochester MN USA

**Keywords:** automatic tube potential, image quality, pediatric abdominopelvic CT, ‘Plan‐Do‐Study‐Act’ quality improvement, radiation dose reduction

## Abstract

It is important to employ radiation dose reduction techniques in pediatric computed tomography (CT) to reduce potential risks of radiation‐induced malignancy. Automatic tube potential (kV) selection tools have been developed and become available on many CT scanners, which select the optimum kV based on the patient size and clinical task to improve the radiation dose efficiency. However, its use in pediatric CT has been mostly empirical, following manufacturer’s default recommendation without solid demonstration for quality improvement. This study aimed to implement an automatic tube potential tool (CAREkV, Siemens Healthcare) into routine pediatric CT practice, using the “Plan‐Do‐Study‐Act” quality improvement process, in place of an existing kV/mAs technique chart. The design of this quality improvement project involved Plan‐Do‐Study‐Act stages. ***Plan*** and ***Do*** stages identified the criteria for optimal automatic kV selection; *a* range of phantoms representing typical pediatric groups were scanned on a dual‐source 128‐slice scanner using a fast‐pitch scanning mode. The identified CAREkV settings were implemented into the CT protocol and evaluated after a 6‐month period. In the ***Study* stage**, an objective evaluation of the image metrics and radiation dose for two similar patient cohorts using CAREkV and the technique‐chart, respectively, were compared. The kV selected, image quality and radiation dose determined by CAREkV were comparable to those obtained while using the technique‐chart. The CAREkV was successfully implemented into our pediatric abdominopelvic CT practice. By utilizing the “***PDSA***” process optimal image quality and radiation dose reduction were achieved with an automatic kV selection tool to improve CT workflow.

## 1 INTRODUCTION

**Fig 1:**
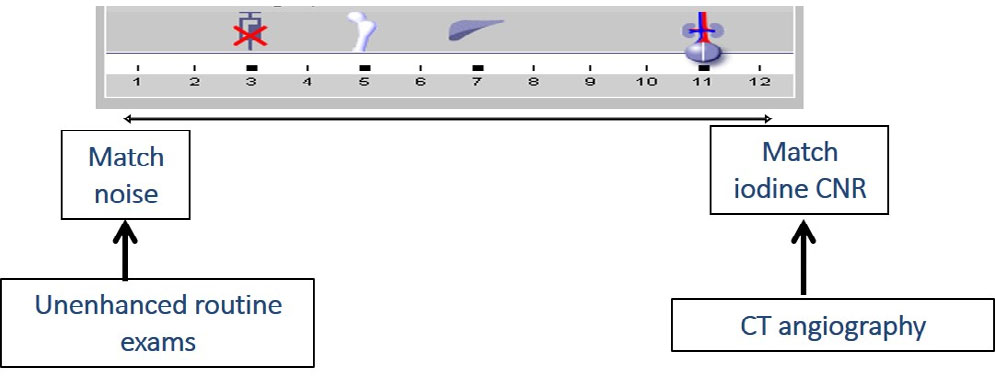
CarekV Slider bar for selecting different diagnostic tasks.

Computed tomography (CT) is an important imaging technique for the detection and staging of disease across the abdominopelvic region of pediatric patients, largely due to the excellent image quality and also due to the fast speed of image acquisition.^1^, ^2^ When imaging a pediatric population with ionizing radiation, it is important that dose reduction techniques are employed to optimize data acquisition and reduce potential risks associated with radiation exposure. To date, a number of optimization tools for radiation dose reduction have been successfully implemented,^3^, ^4^ including automatic tube current modulation^5^ and iterative reconstruction.^6^ Another important technique is tube potential (kV) optimization. It was demonstrated that lower kV may improve the iodine contrast enhancement and contrast‐to‐noise ratio (CNR), especially for relatively smaller patients, including pediatric patients.^7^, ^8^ However, it was also shown that lower kV may increase image noise for relatively larger sized patients. Therefore, technique charts of kV and mAs were commonly used to achieve the benefit of lower kV for smaller patients, while avoiding the drawback of lower kV for bigger patients.^9^, ^10^, ^11^ Despite the success of manual technique chart for radiation dose reduction, the manual process of selecting the technique factors by technologists and entering them onto the scanner for each patient is tedious, error‐prone, and sometimes inaccurate. To resolve this significant issue, an automatic method was developed to allow the most dose efficient kV to be determined in a quantitative manner, which is dependent on both the patient size and the intended diagnostic task.^12^ CAREkV, an automatic kV tool developed by Siemens, selects the optimum kV based on both the patient size and the diagnostic task, optimizing both the radiation dose and the image quality.^13^ Figure 1 illustrates how CAREkV is adjusted using different strength settings of automatic tube potential selection. This strength selection is adjusted depending on diagnostic task and can be adjusted between matching noise and matching iodine CNR, as can be seen in Fig. 1. With the change of this parameter, the strength can be anywhere between matching noise or iodine CNR. For non‐contrast examinations the strength is weaker, adjusted to match noise and there is low probability of selecting a lower tube potential and there is minimal or no dose reduction at this setting. When the strength selection matches iodine, the strength is higher and there is a higher chance of selecting a lower tube potential and there is more potential for dose reduction. The strength setting is specific for each diagnostic task (or protocol) and can be selected differently for pediatric and adult protocols. Once this setting is determined, the tube potential selection and the corresponding radiation dose reduction are fully automatic for different patient sizes. Similar automatic kV selection techniques were commercially implemented by different CT manufacturers (CAREkV, Siemens Healthcare; kV Assist, GE Healthcare). Implementation of CAREkV has been shown to achieve up to 50% dose reduction in adult body CT.^13^, ^14^, ^15^, ^16^

Automatic kV selection is an effective tool for radiation dose reduction. However, clinical implementation of this tool in pediatric CT is not a trivial task; a quantitative approach is required to determine the optimal settings for different clinical tasks which takes into account contrast enhancement, patient size, image quality, and scanning speed.^17^ The aim of this study was to implement an automatic kV selection tool (CAREkV, Siemens) for improved workflow, safety, and image quality with optimized radiation dose in pediatric abdominopelvic CT examinations, using the PDSA quality improvement process. This process involved a combination of phantom studies to identify optimum CAREkV settings and analysis of clinical data to evaluate the impact of implementing CAREkV into the CT protocol. In order to achieve effective and efficient implementation of clinical practice improvement through the use of the CARE kV tool, a data‐driven method which incorporated ongoing evaluation to ensure quality management of the changes was used. This involved the use of the PDSA quality improvement process to provide a systematic approach to implementing clinical practice quality improvements.

## 2 MATERIALS AND METHODS

**Fig 2:**
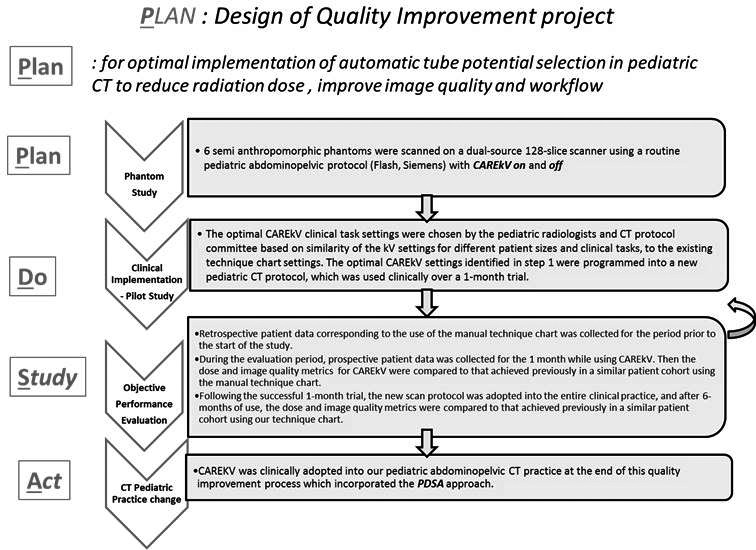
Flow chart of approach taken to incorporate the CAREkV tool into routine pediatric abdominopelvic computed tomography practice in our institution using the Plan‐Do‐Study‐Act process.

Our institutional review board approved the study (IRB 20‐003217); informed consent was not required. Using repeated plan‐do‐study‐act cycles, we subscribed to a 5‐step process for implementation of the CAREkV tool into our pediatric abdominopelvic CT practice for improved image quality and optimized dose (Fig. 2). A radiology quality improvement team was established, which consisted of medical physicists, CT technologists and pediatric radiologists to analyze and monitor the implementation process from September 2018 until April 2019.

### 2.A Step 1‐pre‐implementation planning

**Fig 3:**
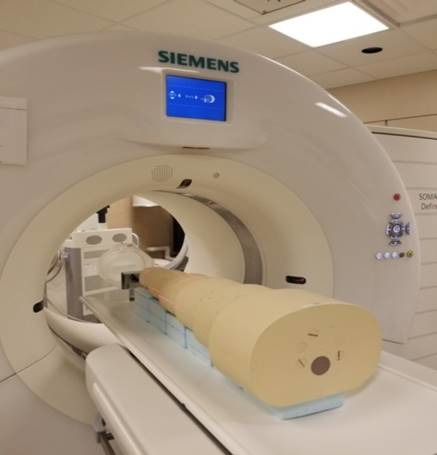
Phantom set‐up of the six semi‐anthropomorphic abdominal phantoms representing different pediatric age groups. Variable height inserts (blue objects in the photograph) were used to achieve the same centering of each phantom.

Prior to the implementation of the CAREkV dose reduction tool, performance metrics were defined which allowed for an assessment of the impact of CAREkV into routine pediatric abdominopelvic CT examinations. These performance metrics were CTDI_vol_, image noise (measured at subcutaneous fat and liver), and iodine CNR (measured on aorta at the level of the top of the liver) and were measured on a range of pediatric patients of different age groups before and after CAREkV was implemented. In order to determine the optimized parameters for CAREkV, the “Do” phase had two sub‐phases: a phantom study and a clinical implementation pilot study. The phantom study was carried out to determine optimal parameter CAREkV settings. Six semi‐ anthropomorphic phantoms (CIRS, Model 007TE) representing the sizes of a newborn, 1‐, 5‐, 10‐, 15‐yr‐old, and young adult, were scanned on a dual‐source 128‐slice scanner (Flash, Siemens) using two different protocols, one with an optimized technique chart (Table 1) where CAREkV was off, and the other with CAREkV turned on. The experimental set‐up is presented in Fig. 3. The water equivalent diameter (WED) of each of the phantoms was measured from the CT image using the following equation:

(1) $$WED\left(\mathrm{cm}\right)=2\sqrt{A_{w}/\pi }=2\sqrt{\frac{\left[\frac{1}{1000}mean\;CT{\left(x,y\right)}_{\mathrm{ROI}}+1\right]A_{\mathrm{ROI}}}{\pi }}$$

where *A_w_* is the attenuation of water normalized in terms of CT numbers, mean *CT(x,y)_ROI_* is the mean CT number in a region of interest (ROI), and *A_ROI_* is the total area of the ROI.^18^

**Table 1:** Technique chart used in pediatric abdominopelvic CT exams which provide the kV and QRM to be employed for pediatric abdominopelvic CT exams without IV‐contrast and with IV‐contrast for three categories of patient weights.

	<8 kg		8–30 kg		>30 kg	
	kV	*QRM* (mAs)	kV	*QRM* (mAs)	kV	*QRM* (mAs)
With IV‐contrast	80	460	100	220	160	160
Non‐contrast	120	160	120	160	120	160

The technique chart was previously developed as an outcome of a comprehensive clinical study.^9^ The patient weight was used for determining the most appropriate technique (kV and QRM) for contrast and non‐contrast CT examinations. In order to link this technique chart and the corresponding patient data with those from a phantom study the following steps were taken. Firstly, the weight range for the age groups matching the phantoms used in this study, namely, newborn, 1‐, 5‐, 10‐, 15‐yr‐old, and young adult, were identified based on previously reported studies.^7^, ^10^, ^19^ The water‐equivalent‐diameter (WED) and the size specific dose estimate (SSDE) were determined for 10 patients corresponding to each age group (n = 60), who underwent abdominopelvic CT examinations using this technique chart in our institution, in order to determine the average WED and SSDE for each patient age group. The WED and SSDE were calculated based on the AAPM Report 204.^20^ This meant that each age group had a range of weights and WEDs which could then be linked to the phantom data through the phantom WED. For the evaluation of CAREkV, six of the slider bar settings corresponding to different clinical tasks were used (namely, 2, 3, 5, 6, 8 and 11) for a reference technique of 120 kV and 160 quality reference mAs (QRM). The kVs and corresponding radiation doses (CTDI_vol_) were recorded for each phantom size and each clinical task setting.

### 2.B Step 2‐Clinical implementation — pilot trial of CAREkV

The optimal CAREkV settings identified were programmed into a new pediatric CT protocol on one CT scanner (Flash, Siemens), which was evaluated in a pilot trial over a 1‐month period. During this clinical evaluation period, various patient data were recorded, including: patient size [as measured by water‐equivalent‐diameter (WED)]; clinical task; the kV and CTDI_vol_ selected by CAREkV; and the average subcutaneous fat noise, average liver noise, and the aortic iodine CNR as image quality metrics.

### 2.C Step 3‐objective performance evaluation — pilot trial of CAREkV

The dose and image quality data for the CAREkV tool for this 1‐month pilot trial (n = 25) were then compared to that achieved previously in a similar patient cohort that used the manual technique chart (n = 25).

### 2.D Step 4‐objective performance evaluation — full practice implementation of CAREkV

Following the successful 1‐month trial, the new scan protocol was adopted into the entire clinical practice, and after 6‐months of use (n = 56), the dose and image quality metrics were compared to that achieved previously in a similar patient cohort using the manual technique chart (n = 60).

### 2.E Step 5 – adoption of CAREkV

The CAREkV tool was clinically adopted into our pediatric abdominopelvic CT practice at the end of this quality improvement process which incorporated the ***PDSA*** approach.

### 2.F Statistical analysis

When applicable, descriptive statistics were employed, and data are presented as means ± standard errors of the mean. Differences in group data were considered significant at *p* < 0.05. Differences between the medians of the two independent groups (i.e., the number of cases submitted per month prior to implementation and the number of cases submitted after implementation) were compared by using the Student’s *t*‐test. Analyses were performed using JMP software (JMP IN 5.1; SAS Institute, Cary, NC). A comparison was made between the patients in the technique chart group and the patients in the CAREkV *on* group.

## 3 RESULTS

### 3.A Step 1‐pre‐implementation planning

**Fig 4:**
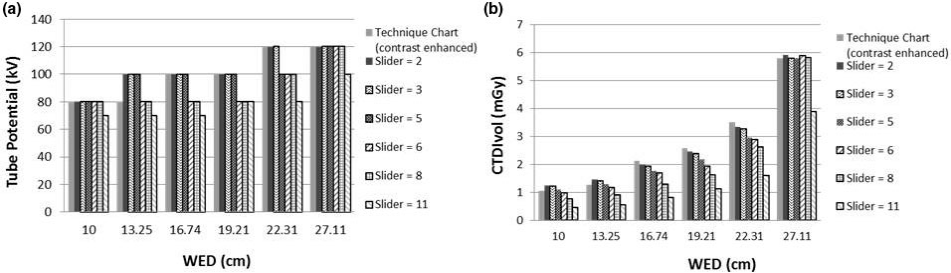
(a) kV selection and (b) radiation dose (CTDI_vol_) used for the different phantom sizes and slider‐bar settings.

Using the six semi‐anthropomorphic phantoms, the range of tube potential selected by the CarekV technique and the CTDI_vol_ measured for each phantom size and diagnostic task, as defined by the slider bar setting, are presented in Fig. 4. The optimal CAREkV clinical task setting was chosen by the radiology quality improvement team and CT protocol committee based on similarity of the kV settings for different patient sizes and clinical tasks, to the existing technique chart settings. It was determined that the kVs selected by CAREkV, for the settings of 2 and 5, were potentially the most suitable for non‐contrast and contrast examinations, respectively (Table 2).

**Table 2:** CAREkV Recommended kV ‐ Summary of tube potential selection by CAREkV for the different phantom sizes and slider‐bar settings.

WED (cm)	Newborn 10 cm		1 yr 13.25 cm	5 yr 16.74 cm	10 yr 19.21 cm	15 yr 22.31 cm	Young adult 27.11 cm
Non‐contrast clinical task ‐ Slider 2	80		100	100	100	120	120
Slider 3	80		100	100	100	100	120
Slider 5	80		100	100	100	100	120
Slider 6	80		80	80	80	100	120
Slider 8	80		80	80	80	100	120
IV contrast clinical task ‐ Slider 11	70		70	70	80	80	100

### 3.B Step 2‐clinical implementation — pilot trial of CAREkV

The optimal CAREkV settings identified in Step 1 were programmed into a new pediatric CT protocol, and used clinically for a 1‐month trial.

### 3.C Step 3‐Objective performance evaluation — pilot trial of CAREkV

At the end of the 1‐month trial using one CT scanner, a retrospective survey was carried out evaluating the kV selected by CAREkV and measured radiation dose, CTDI_vol_ for all the pediatric patients who underwent an abdominopelvic CT examination. An analysis of the selected tube voltage and measured CTDI_vol_ as a function of patient size, determined as water‐equivalent‐diameter, was carried out and the results are presented in Table 3. All of the cases analyzed were contrast‐enhanced examinations, so a CAREkV slider bar setting of “5” was selected and utilized. It was found that both the tube potential selected and radiation dose determined by CAREkV were comparable to those obtained when the technique chart was used. Furthermore, the image quality was monitored by the pediatric radiologists during the pilot trial and was found to be satisfactory. It was decided to conduct a larger study using all Flash CT scanners in our institution to fully evaluate the implementation of CAREkV into pediatric abdominopelvic CT examinations.

**Table 3:** Comparison of patient‐averaged Water Equivalent Diameter (WED), CTDIvol and Size Specific Dose Estimate (SSDE) with the technique chart (Group 1) and CarekV (Group 2) for different patient sizes in abdominopelvic CT.

Age group	Number of patients	Technique chart			CAREkV ON 1‐month (step 3)				CAREkV ON 6‐month (step 4)			
		Patient‐averaged WED (cm) ± STD	Patient‐averaged CTDI_vol_ (mGy) ± STD	Size specific dose Est (mGy) ± STD	Number of patients	Patient‐averaged WED (cm) ± STD	Patient‐averaged CTDI_vol_ (mGy) ± STD	Size specific dose est (mGy) ± STD	Number of patients	Patient‐averaged WED (cm) ± STD	Patient‐averaged CTDI_vol_ (mGy) ± STD	Size specific dose est (mGy) ± STD
Newborn	10	13.0 ± 1.9	1.3 ± 0.3	3.0 ± 0.3	4	15 ± 1.2	2.3 ± 0.6	5.0 ± 1.3	8	14.3 ± 1.2	1.7 ± 0.7	3.9 ± 1
1 year	10	15.6 ± 2.0	2.6 ± 0.8	5.4 ± 1.4	5	16.7 ± 1.2	2.4 ± 0.6	5.2 ± 1.3	10	17.8 ± 1.3	2.0 ± 0.7	5 ± 0.9
5 years	10	17.9 ± 3.1	4.1 ± 1.2	7.8 ± 2	4	20.7 ± 2.9	3.3 ± 1	6.2 ± 1.3	9	21.5 ± 2.3	4.4 ± 1.4	7.5 ± 1.7
10 years	10	21.2 ± 4.5	5.1 ± 1.1	9.5 ± 2.3	5	23.3 ± 2.3	5.1 ± 1.6*	8.8 ± 2.2*	9	24.4 ± 1.8	5.7 ± 1.6*	9.3 ± 2.4*
15 years	10	22.1 ± 6.0	6.3 ± 1.0	9.7 ± 3.6	6	24.9 ± 1.5	6.2 ± 0.7	9.5 ± 1	10	25.3 ± 2.5	6.3 ± 1.0	9.9 ± 1
18 years	10	26 ± 6.0	8.5 ± 4.4	11 ± 4.9	6	25.3 ± 1.5	7.4 ± 1.3	11.1 ± 2.0	10	27.7 ± 3.5	8.2 ± 2.8	10.1 ± 2.4

*Three pear shaped patients were included in this data analysis and these patients were scanned using a tube potential of 140kV. CAREkV prescribed a 140 kV because the required tube current at 120 kV exceeded the limit at the high helical pitch of 3 which is employed for pediatric scanning and thus the tube potential was increased to meet the required radiation dose.

### 3.D Step 4‐objective performance evaluation — full practice implementation of CAREkV

**Fig 5:**
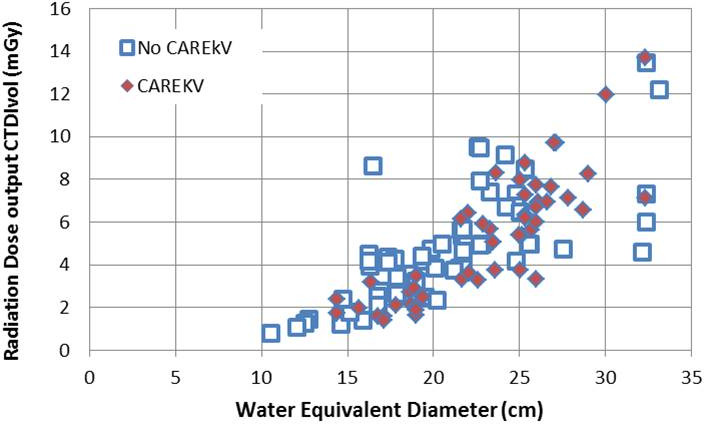
CTDI_vol_ of patients of different sizes with the use of the technique chart (No CAREkV, n = 60) and ***with*** the use of CAREkV (CAREkV, n = 56).

A comparison of the selected tube potential, CTDI_vol_ and image metrics for the technique chart and the implemented CAREkV tool was carried out through retrospective analysis of abdominopelvic examinations for both. Both groups were found to have similar patient cohorts (*p* = 0.4) based on the Student’s t‐test analysis of the two groups of patient‐averaged WEDs (the patient average weight for each age group can be seen in Table 3). It was found that the CTDI_vol_ measurements for different patient sizes and clinical tasks after the implementation of the CAREkV tool were comparable to those for the technique chart (Fig. 5), with no statistically significant difference (*p* = 0.4).

**Fig 6:**
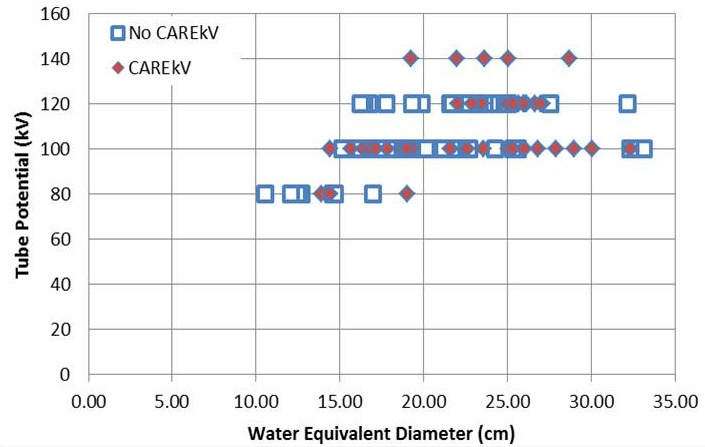
kV used for patients of different sizes and clinical tasks with the use of the technique chart (No CAREkV, n = 60) and ***with*** the use of CAREkV (CAREkV, n = 56); both groups had similar patient cohorts (comparison between WEDs from each group, *P* = 0.4).

**Fig 7:**
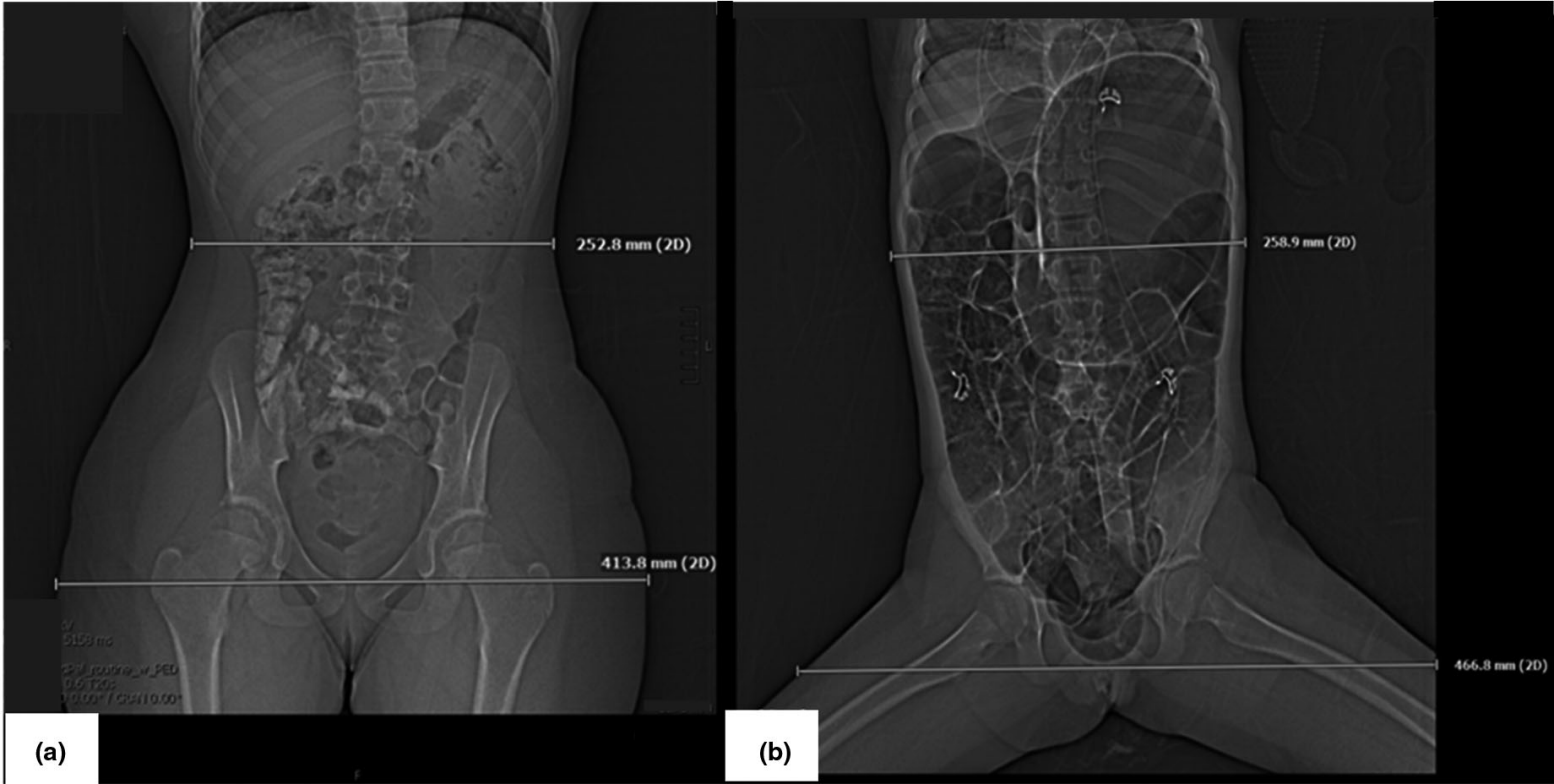
(a) Topogram of one of the pear shaped patients demonstrating the difference between the width at the level of the top of the liver (253 mm) compared to the level at the hips (414 mm) and (b) patient with non‐optimum positioning at the level of the top of the liver (259 mm) compared to the level at the hips (467 mm).

The tube potential was optimally selected for the majority of cases apart from five patient cases where the CAREkV tool selected a higher tube potential of 140 kV (Fig. 6). The reason a higher kV setting was selected was due to three patient’s ***pear shape*** body habitus (i.e., wider hips than abdomen, Fig. 7), one was very large extending beyond the Field‐of‐View, and one had a ***non‐optimum*** set‐up (legs apart). The wide anatomy thus presented by these patients caused the kV to be increased to 140 kV as there ***was not sufficient tube output*** at the ***higher pitch*** used for the pediatric flash CT imaging examinations. In order to maintain the required output, the higher kV was selected as the tube current was maxing out at the selected pitch at 120 kV. This is an indication that the WED as measured at one anatomical location does not always provide a good representation of patient size, in particular, for the hip region of pear‐shaped patients. Following this analysis of the data, iteration was carried out in the study, changing the implemented protocol to block the use of this higher tube potential of 140 kV by CAREkV for pediatric abdominopelvic CT examinations, thereby reducing the dose and providing better contrast in this type of examination in the future.

**Fig 8:**
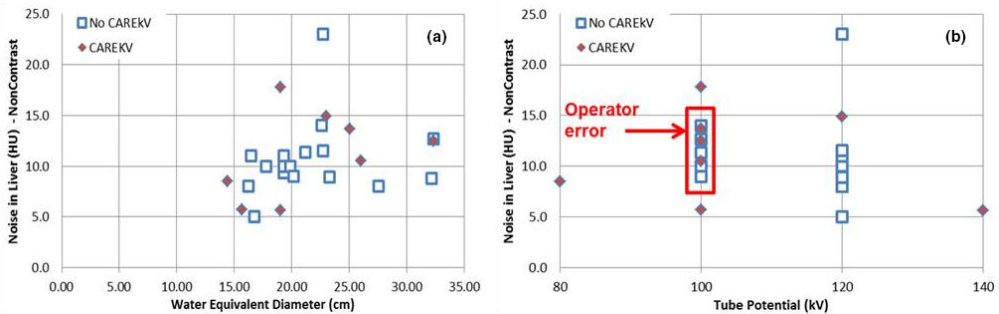
Noise in liver for **non‐contrast exams** as a function of (a) patient size and (b) kV selected for the patient, with the use of the technique chart (No CAREkV, n = 16) and ***with*** the use of CAREkV (CAREkV, n = 8).

The analysis of the image metrics revealed that there was no significant difference (*p* = 0.8) between the image quality of the examinations performed using the technique chart and CAREkV, specifically, **noise in liver** for non‐contrast exams (Fig. 8). An interesting and unexpected finding was that 42% of analyzed non‐contrast examinations protocoled using the technique chart were incorrectly setup using a tube potential of 100 kV rather than 120 kV, indicating the ***presence of operator‐error*** in the CT protocol setup. The implementation of the CAREkV tool into routine clinical practice removed this potential for operator error in addition to providing a better workflow.

**Fig 9:**
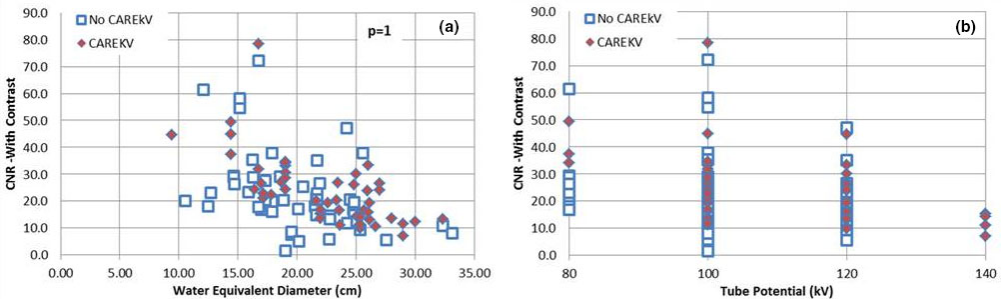
Contrast to noise ratio (CNR) for **contrast‐enhanced exams as a function of** (a) patient size and (b) kV selected for the patient, with the use of technique chart (No CAREkV, n = 44) and ***with*** the use of CAREkV (CAREkV, n = 48).

Furthermore, it was found that there was no significant difference (*p* = 1) between the CNR for contrast‐enhanced exams between the two groups (Fig. 9).

### 3.E **Step 5 – adoption of CAREkV** — **Act: clinical implementation of CAREkV into routine pediatric abdominopelvic CT exams**

Following a 6 month PDSA study, the optimal CAREkV clinical task setting for different patient sizes and clinical tasks were identified and programmed into the pediatric CT protocol for abdominopelvic CT examinations. The implementation of this dose reduction technique required the use of the PDSA approach to maintain (or decrease) the dose while maintaining the image quality through the systematic evaluation of the dose and image quality metrics. The implementation of dose reduction techniques routinely utilized in adult CT cannot be directly transferred into pediatric CT without appropriate evaluation, as the noise level which is deemed acceptable in adult examinations is not regarded as acceptable in pediatric examinations. Consequently, these dose reduction techniques cannot be directly transferred into pediatric CT examinations.^3^, ^20^, ^21^ The use of the automatic tube potential tool, CAREkV, also eliminates the probability of human error occurring in scan protocol setup, and speeds up the workflow. The PDSA involved a single iteration in the cycle based on the results from the objective performance evaluation; namely, the CAREkV CT protocol was further refined to exclude the selection of a tube potential of 140 kV, thereby providing the benefits of improved contrast and dose reduction at lower tube potentials. Scanning speed is an important factor that must be considered in pediatric examinations, and a tradeoff typically exists between the scanning speed and the maximum achievable radiation dose. CT systems limit the tube current to prevent the tube overload at high pitches which are typically used in pediatric CT exams, and increased the tube voltage to 140 kV compensate for the tube overload. This new pediatric CT protocol has been accepted for routine pediatric abdominopelvic exams and will be reviewed annually as part of CT protocol management program.

## 4 DISCUSSION AND CONCLUSIONS

A PDSA project was setup to implement CAREkV into our pediatric abdominopelvic CT practice. In order to achieve this, the first phase of the study was a phantom investigation comparing the use of the current weight‐based technique chart with CAREkV; this phantom data to our previously established weight‐based dose chart. Optimal settings for an automatic tube potential selection tool were determined for different patient sizes. The results from the phantom study were validated against retrospective examinations which utilized an optimized technique chart determined from a comprehensive clinical evaluation. Optimal slider bar settings for a range of patient sizes and both non‐contrast and contrast abdominal examinations were selected by experienced users from the phantom data, which most closely matched those implemented by the weight‐based technique chart and used in a 1‐month trial using CarekV as the pediatric abdominopelvic CT protocol. Following the one‐month trial, the data from those pediatric abdominopelvic CT examinations were analyzed and compared to match technique chart examinations acquired before the trial. Once this was validated and shown to have no significant impact on image quality and dose (CTDI_vol_), CAREkV was implemented as our pediatric abdominopelvic CT standard practice.

It should be noted that implementation of dose reduction techniques routinely utilized in adult CT cannot be directly transferred into pediatric CT without appropriate evaluation, as the noise level which is deemed acceptable in adult examinations is not regarded as acceptable in pediatric examinations. Direct extrapolation of adult CAREkV to pediatric patients may have picked a higher strength CAREkV setting, for example, the manufacturers default is between 7 and 11. This would have had higher noise associated with lower tube potential. Therefore, direct conversion from existing well‐established technique chart is essential to make sure diagnostic quality is not sacrificed with the introduction of automatic tube potential selection.

In summary, CAREkV was clinically adopted into our pediatric abdominopelvic CT practice with use of the 5‐step procedure. This ensured that optimal image quality was maintained relative to our technique chart rigorously‐developed in a previous clinical study, and that an appropriate radiation dose reduction was incorporated by the CAREkV tool through careful selection of the clinical task parameter settings. This tool provides the benefits of radiation dose reduction, streamlined workflow, and reduction in human‐error in the protocol set‐up while maintaining the requisite image quality.

## CONFLICT OF INTEREST

Dr. C.H. McCollough received industry funding from Siemens Healthcare. The other authors have no conflicts to disclose.

## 5 AUTHOR CONTRIBUTIONS

JEB was involved in data collection and analysis as well as manuscript preparation. MRB and VT were involved in data collection. KRT, NCH and CHM were involved in data analysis and manuscript review. LY was involved in study design, data analysis and manuscript preparation. The authors declare that they had full access to all of the data in this study and the authors take complete responsibility for the integrity of the data and the accuracy of the data analysis.

## ETHICS APPROVAL

Our institutional review board approved the study (IRB 20‐003217); informed consent was not required).
